# Supplementary material for: A UMLS-based spell checker for natural language processing in vaccine safety
Source: BMC Med Inform Decis Mak. 2007 Feb 12;7:3. doi: 10.1186/1472-6947-7-3 (PMC1805499; doi:10.1186/1472-6947-7-3)
Supplement: Additional file 13 — RAP application directory. Sets up directory for RAP (RDF application for PHP) source code [file 1472-6947-7-3-S13.gz › rap/test/test_setoperations.php]

Test Set Operations

php
define("RDFAPI\_INCLUDE\_DIR", "./../api/");
include(RDFAPI\_INCLUDE\_DIR . "RdfAPI.php");
echo "<h31. Generate and show two MemModels";
// Create empty MemModel
$model = new MemModel();
$model->setbaseURI("http://www.bizer.de");
$model2 = new MemModel();
$model2->setbaseURI("http://www.bizer.de/zwei");
// Create nodes and add statements to models
$myhomepage = new Resource("http://www.bizer.de/welcome.html");
$creator = new Resource("http://purl.org/dc/elements/1.1/creator");
$me = new Resource("mailto:chris@bizer.de");
$model->add(new Statement($myhomepage, $creator, $me));
$model2->add(new Statement($myhomepage, $creator, $me));
$creation\_date = new Resource("http://www.example.org/terms/creation-date");
$August16 = new Literal("August 16, 2002");
$model->add(new Statement($myhomepage, $creation\_date, $August16));
$model2->add(new Statement($myhomepage, $creation\_date, $August16));
$language = new Resource("http://www.example.org/terms/language");
$deutsch = new Literal("Deutsch", "de");
$model->add(new Statement($myhomepage, $language, $deutsch));
$name = new Resource("http://www.example.org/terms/Name");
$chrisbizer = new Literal("Chris Bizer");
$model2->add(new Statement($me, $name, $chrisbizer));
// Output as Table
echo "

##### Model 1

";
$model->writeAsHtmlTable();
echo "

##### Model 2

";
$model2->writeAsHtmlTable();
echo "

";
echo "

### 2. Make some tests

";
echo "Test: Model 2 contains any statements from model 1 :".$model2->containsAny($model)."

";
echo "Test: Model 1 contains any statements from model 2 :".$model->containsAny($model2)."

";
echo "Test: Model 2 contains all statements from model 1 :".$model2->containsAll($model)."

";
echo "Test: Model 1 contains all statements from model 2 :".$model->containsAll($model2)."

";
echo "Test: Model 1 equals model 2 :".$model->equals($model2)."

";
echo "

### 3. Unite model 1 and model 2

";
$model3 =& $model->unite($model2);
$model3->writeAsHtmlTable();
echo "

### 4. Intersect model 1 and model 2

";
$model4 =& $model->intersect($model2);
$model4->writeAsHtmlTable();
echo "

### 5. Substract model 2 from model 1

";
$model5 =& $model->subtract($model2);
$model5->writeAsHtmlTable();
echo "

### 6. Reify model 1

";
$model6 =& $model->reify();
$model6->writeAsHtmlTable();
?>
